# BRD4 associates with p53 in DNMT3A-mutated leukemia cells and is implicated in apoptosis by the bromodomain inhibitor JQ1

**DOI:** 10.1002/cam4.146

**Published:** 2013-10-31

**Authors:** Helen Jayne Susan Stewart, Gillian Abigail Horne, Sarah Bastow, Timothy James Telfer Chevassut

**Affiliations:** 1 Brighton and Sussex Medical School, University of SussexBrighton, East Sussex, BN1 9PS, U.K; 2 Department of Haematology, Royal Sussex County HospitalBrighton, East Sussex, BN2 5BE, U.K

**Keywords:** AML, BRD4, bromodomain, DNMT3A, JQ1, p53

## Abstract

The bromodomain and extra terminal (BET) family protein bromodomain containing protein 4 (BRD4) is an epigenetic regulator recently identified as a therapeutic target for several hematological cancers, notably mixed lineage leukemia-fusion acute myeloid leukemia (MLL-AML). Here, we show that the BRD4 bromodomain inhibitor JQ1 is highly active against the p53-wild-type Ontario Cancer Institute (OCI)-AML3 cell line which carries mutations in nucleophosmin (NPM1) and DNA methyltransferase 3 (DNMT3A) genes commonly associated with poor prognostic disease. We find that JQ1 causes caspase 3/7-mediated apoptosis and DNA damage response in these cells. In combination studies, we show that histone deacetylase (HDAC) inhibitors, the HDM2 inhibitor Nutlin-3, and the anthracycline daunorubicin all enhance the apoptotic response of JQ1. These compounds all induce activation of p53 suggesting that JQ1 might sensitize AML cells to p53-mediated cell death. In further experiments, we show that BRD4 associates with acetylated p53 but that this association is not inhibited by JQ1 indicating that the protein–protein interaction does not involve bromodomain binding of acetylated lysines. Instead, we propose that JQ1 acts to prevent BRD4-mediated recruitment of p53 to chromatin targets following its activation in OCI-AML3 cells resulting in cell cycle arrest and apoptosis in a c-MYC-independent manner. Our data suggest that BET bromodomain inhibition might enhance current chemotherapy strategies in AML, notably in poor-risk DNMT3A/NPM1-mutated disease.

## Introduction

Several recent papers have demonstrated the therapeutic potential of targeted inhibition of bromodomain-containing proteins in certain hematological malignancies including acute myeloid leukemia (AML) [1, 2] and multiple myeloma (MM) [3, 4]. The target proteins belong to the bromodomain and extra terminal (BET) family of adaptors (BRD2, BRD3, BRD4, and BRDT), which contain acetyl-lysine recognition motifs, or bromodomains, that “read” posttranslational acetylation modifications of chromatin [5, 6] and other proteins modified by acetylation such as nuclear factor kappa light chain enhancer of activated b cells (NFκB) [7].

BRD4 in particular has been identified as a key therapeutic target in AML based on an RNAi screen of epigenetic genes in an mixed lineage leukemia (MLL) fusion leukemia mouse model [1]. The bromodomains of the BET family proteins bind acetylated lysine residues present in histone tails that are typically associated with an open chromatin state and transcriptional activation [8]. Small molecule inhibitors of the bromodomain pocket, such as JQ1 and I-BET, disrupt bromodomain containing protein 4 (BRD4) recruitment to chromatin leading to downregulation of key oncogenes, notably cellular-myelocytomatosis oncogene (c-MYC) [1, 3, 4], BCL2, and CDK6 [2]. This results in reactivation of the p21 tumor suppressor [4] leading to cell cycle arrest and apoptosis of many, but not all, hematological cell lines and malignant primary cells, particularly those with MLL gene rearrangements. JQ1 interferes with the ability of BRD4 to “read” acetylated histones that facilitate transcriptional activation.

Although the effects of BRD4 inhibition are at least partially due to its role in sustaining c-MYC expression, the downregulation of c-MYC induced by JQ1 is not sufficient to cause apoptosis as c-MYC downregulation has been observed in cell lines that are poorly responsive to JQ1 such as K562 cells [1]. Furthermore, ectopic c-MYC expression is unable to prevent JQ1-induced cell death but can overcome cell cycle arrest and differentiation induced by this compound [1].

To our knowledge, this study is the first to show that JQ1 is active against the Ontario Cancer Institute (OCI)-AML3 cell line which carries mutations of the nucleophosmin (NPM1) and DNA methyltransferase 3 (DNMT3A) genes that are highly recurrent in AML and commonly associated with poor risk disease. Beyond this, the principal aim of the study was to explore the broader therapeutic potential of BET bromodomain inhibitors by identifying synergistic interactions with other compounds and to probe the mechanism of JQ1 action. We show that combined treatment with other compounds known to activate p53, namely histone deacetylase (HDAC) inhibitors, Nutlin-3 and daunorubicin, all potentiate the action of JQ1 on OCI-AML3 cells suggesting JQ1-induced cell death is via a p53-mediated pathway that appears to involve a caspase 3/7-dependent mechanism. Furthermore, we show that BRD4 associates with activated p53 suggesting that BRD4 inhibition by JQ1 may result in failed recruitment of p53 to chromatin leading to impaired DNA damage repair response, cell cycle arrest, and cell death.

## Material and Methods

### Reagents

Vorinostat was from Stratatech (Suffolk, U.K.), Nutlin-3, trichostatin A and sodium butyrate were obtained from Sigma (Poole, U.K.); penicillin, streptomycin, l-glutamine and RPMI 1640, TRIzol and reverse transcription polymerase chain reaction (RT-PCR) primers were purchased from Invitrogen (Paisley, U.K.), fetal calf serum was from Biosera (Ringmer, U.K.). Celltiter-Glo, Caspase–Glo8 and Caspase-Glo 3/7 kits were from Promega (Southampton, U.K.). Anti-γH2AX antibody was from Upstate Biotechnology (Watford, U.K.) and anti-53BP1 and anti-BRD4 were from Cambridge Biosciences (Cambridge, U.K.). Anti-p53 and anti-c-MYC were from New England Biolabs (Hitchin, U.K.). Complete™ protease inhibitor cocktail (Roche, Burgess Hill, U.K.) (ethylenediaminetetraacetic acid [EDTA]-free) were from Roche (Burgess Hill, U.K.). Annexin V/propidium iodide apoptosis detection kit was from BD Biosciences (Oxford, U.K.). Protein A/G-Sepharose beads were from GE Healthcare (Chalfont St Giles, U.K.).

### Leukemia cell lines

Human Leukemia OCI-AML3 (AML-M4 subtype; DNMT3A-R882; NPM1c-mutated; p53-wildtype) and K562 (breakpoint cluster-Abelson murine leukemia viral oncogene homolog 1, BCR-ABL) and Lymphoma RAJI (Burkitt, MYC) cell lines were kind gifts from Dr. T. Gaymes (Kings College, London, U.K.) [9]. Cells were cultured in RPMI 1640 supplemented with 10% fetal calf serum, 100 mmol/l l-glutamine, penicillin (100 IU/mL), streptomycin (100 μg/mL). The identity of the OCI-AML3 cell line was confirmed by carrying out a restriction-sensitive PCR assay for mutated DNMT3A at codon R882 which this cell line harbors [10, 11].

### Growth curves

OCI-AML3 cells were plated at a density of 80,000 cells per well in a 24-well plate in 400 μL of cell culture medium in the presence or absence of 0.25 μmol/L JQ1. Phosphate-buffered saline (PBS) or JQ1 inactive enantiomer was added to control wells. Cells were counted every 24 h for 96 h in the presence of trypan blue (to determine cell viability) and the results recorded.

### Cell viability assays

For cell lines cell viability was assessed using a WST-1 (4-[3-(4-iodophenyl)-2-(4-nitrophenyl)-2H-5-tetrazolio]-1,3-benzene disulfonate) assay. 2 × 10^4^ cells plated per well in 96-well plates were incubated in the presence of compound for the times indicated. Two to four hours after the addition of WST-1, plates were read at 450 nm in a Biotek Synergy plate reader (Biotek, Potton, U.K.) and Gen5 version 1.08 software. The viability of untreated cells was set as 100%, and viability in other groups was calculated by comparing the optical density readings with the control. All compounds were dissolved in dimethylsulfoxide and stored at −20°C. Dilutions in PBS were used for experiments.

### Flow cytometry

Cells were treated with 0.25 μmol/L JQ1, 2 μmol/L Nutlin-3 or a combination of both for 24 h and then stained with anti-annexin V and propidium iodide using an apoptosis detection kit (Promega, Southampton, U.K.). Labeled cells were analyzed using a BD FACS Canto II six color (with red and blue lasers) flow cytometer (BD, Oxford, U.K.).

### Immunolabeling of γH2AX and 53BP1

Cells were cytospun onto glass slides using a Shandon 3 cytospinner (Thermo Scientific, Northampton, U.K.) for 5 min at 650 rpm. Preparations were fixed in 4% paraformaldehyde for 15 min and then permeabilized with 0.2% Triton X100 for 3 min. Mouse anti-γH2AX (1:500) (Millipore, Watford, U.K.) and rabbit anti-53BP1 (1:1000; Bethyl labs, Cambridge Biosciences, Cambridge, U.K.) were applied for 1 h at room temperature. After washing through PBS, cells were incubated in anti-rabbit Alexa Fluor 488 (Life Technologies, Paisley, U.K.) (1:250) and anti-mouse Alexa Flour 546 (1:250) for 30 min. After a final wash through PBS, the slides were incubated in DAPI (4′,6-diamidino-2-phenylindole) for 5 min and then mounted in Citifluor anti-fade mounting medium and viewed for fluorescence using a Leica DM 5000B microscope fitted with a Leica DPC300FX digital camera (Leica, London, U.K.).

### Caspase 3/7 and caspase 8 assays

Activation of caspase 3/7 and caspase 8 was measured using a Caspase-Glo 3/7 and Caspase-Glo 8 assays, respectively. Cells were plated at a density of 20,000 cells per well in black 96-well plates and treated with 0.5 μmol/L JQ1 for various time points up to 48 h.

### Preparation of cell extracts

Cells were isolated in a cooled centrifuge and washed with 0.5 mL of ice-cold PBS containing 40 mmol/L 2-glycerophosphate and 2 mmol/L benzamidine. Pellets were resuspended in 200 μL of ice-cold buffer A (20 mmol/L Mops/KOH [pH 7.2], 10% [v/v] glycerol, 20 mmol/L sodium fluoride, 1 μmol/L microcystin LR, 75 mmol/L KCl, 2 mmol/L MgCl_2_, 2 mmol/L benzamidine, 2 mmol/L sodium orthovanadate and Complete™ protease inhibitor cocktail [Roche] [EDTA-free]) and lysed by vortex mixing following the addition of 0.5% Nonidet P40 and 0.5% sodium deoxycholate. Cell debris was removed by centrifugation in a microfuge at 10,000 *g* for 5 min at 4°C, and the resultant supernatants were frozen in liquid nitrogen until use. For immunoprecipitation experiments cells were pretreated with 500 nmol/L daunorubicin and 400 nmol/L trichostatin A (TSA) for 24 h to induce p53 expression and hyper-acetylation prior to preparation of cell extracts.

### Immunoprecipitation

Equal amounts of total protein extracts from OCI-AML3 or Hela cells were precleared with protein A/G-Sepharose beads (GE Healthcare) at 4°C for 1 h. Supernatants were immunoprecipitated for 2 h at 4°C with a BRD4-specific antibody or with rabbit immunoglobulin as a negative control. The protein–antibody complexes were pulled down by adding protein A/G-Sepharose beads. Sample pellets were then subjected to several washes in PBS. The immunocomplexes were recovered from the protein A/G-Sepharose beads by boiling the samples in electrophoresis loading buffer. The immunocomplexes were analyzed by Western blot using anti-p53 antibodies.

### Western blotting

OCI-AML3 cells were treated with 0.5 μmol/L JQ1 for 24 h and then harvested. Samples were then diluted in loading buffer (with β-mercaptoethanol), boiled for 5 min, and subjected to gel electrophoresis on 10% sodium dodecyl sulfate-polyacrylamide gels (SDS-PAGE). A quantity of 36 μg of cell extract was loaded per lane. Proteins were electrophoretically transferred onto PVDF for 1 h at 150 mA constant current. Immunolabeling was achieved by blocking the gel with 5% milk for 1 h at room temperature and then rotated overnight at 4°C with primary antibodies diluted 1:2000 in Tris-buffered saline 0.1% Tween20. Secondary antibodies were used at a dilution of 1:20,000. Blots were developed using a Vectastain ABC kit or a chemiluminescent detection kit (Vector labs, Peterborough, U.K.).

### Statistical analysis

All values are shown as mean ± SEM from at least three independent experiments (or a representative experiment of three is shown) and considered significant if *P* < 0.05. Significance between groups was calculated using Student's *t*-tests.

## Results

### JQ1 inhibits proliferation of the AML cell line OCI-AML3 in a dose-dependent manner

The bromodomain inhibitor JQ1 has been reported to inhibit the proliferation of many leukemia cells lines, particularly those containing MLL mutations [1]. We performed experiments on OCI-AML3, a p53-wildtype AML cell line that carries mutations in DNMT3A (R882C) and NPM1c (exon-12) genes [12].

**Figure 1:**
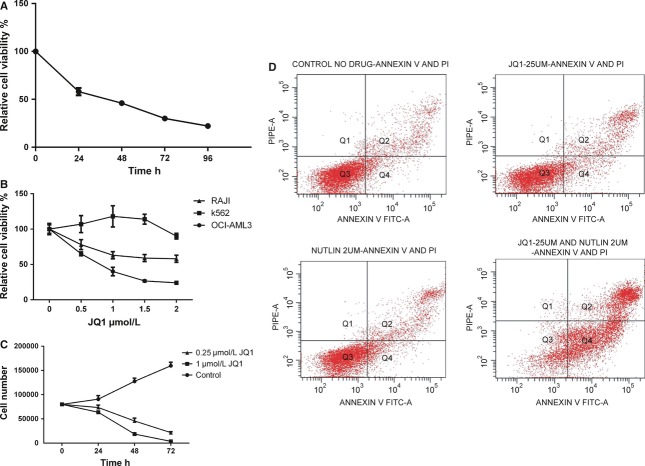
JQ1 mediates cell death in OCI-AML3 cells. (A) OCI-AML3 cells were treated with 1 μmol/L JQ1 for 96 h and then cell viability was measured using the WST-1 assay. (B) A time–response curve for 1 μmol/L JQ1 is shown. (C) Growth curves carried out over a 72-h period in the presence of 0.25 or 0.5 μmol/L JQ1. (D) Cells were treated with 0.25 μmol/L JQ1, 0.5 μmol/L Nutlin-3 or a combination of both for 24 h and stained for FACS analysis. Data are representative of at least three independent experiments performed in triplicate ± SEM.

In preliminary experiments, a time–response curve to analyze the effect of 1 μmol/L JQ1 on OCI-AML3 cell viability was carried out using WST-1 assays. In subsequent experiments, cells were analyzed at 72 h as this treatment time caused a significant decrease in viability (Fig. 1A). We found that JQ1 caused a dose-dependent decrease in cell viability with an IC_50_ of ∼500 nmol/L (Fig. 1B). Control experiments using the inactive enantiomer (–)-JQ1 or vehicle had no effect. For comparative purposes, K562 and Raji cells were used in additional experiments. The p53-mutated K562 cell line responded poorly to the compound as previously reported [1] with an IC_50_ of >2 μmol/L whereas the Raji cell line was moderately sensitive with an IC_50_ of ∼1–2 μmol/L (Fig. 1B).

Growth curves confirmed that the decrease in cell viability observed in JQ1-treated OCI-AML3 cells was due to cell death (Fig. 1C). Flow cytometry analysis using propidium iodide and annexin V staining confirmed that death was by apoptosis (Fig. 1D).

### JQ1 induces double-stranded DNA breaks and pan-nuclear γH2AX staining

**Figure 2:**
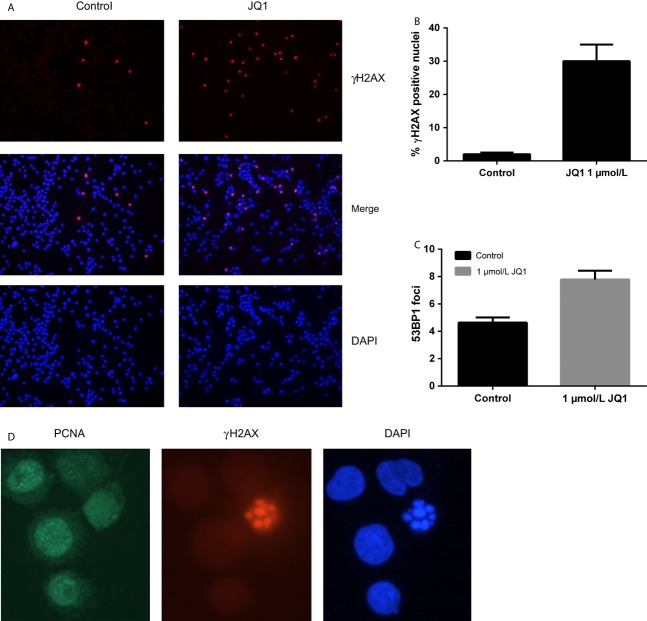
JQ1 induces pan nuclear γH2AX labeling and 53BP1 foci in OCI-AML3 cells. (A) OCI-AML3 cells were treated with 1 μmol/L JQ1 for 24 h and then immunolabeled for γH2AX. (B) Graph showing γH2AX-positive cells (pan-nuclear staining). (C) Graph showing 53BP1 foci (counted under 100× magnification); Results shown are the mean of three separate experiments ± SEM; *P < 0.05 as calculated by Student's *t-*test comparing control and JQ1-treated cells. (D) Cells immunolabeled for PCNA and γH2AX after treatment with 1 μmol/L JQ1.

In order to determine the mechanism of action of JQ1, we carried out experiments to look for DNA damage responses. OCI-AML3 cells treated with 0.25 μmol/L JQ1 were immunolabeled for H2AX phosphorylation and 53BP1. JQ1 induced a robust increase in pan-nuclear γH2AX staining indicative of an early apoptotic response (Fig. 2A and B). This was accompanied by a significant increase in 53BP1 foci (Fig. 2C) but not γH2AX foci. Only 10% of cells with pan-nuclear γH2AX staining had 53BP1 foci. No increase in pan-nuclear γH2AX staining was observed at 48 h although DAPI staining showed an increase in pyknotic nuclei at this stage from 1.5 ± 0.2% to 3.2 ± 0.17% (Fig. 2D, middle panel).

Time course experiments showed that induction of pan-nuclear γH2AX occurred between 7 and 16 h after JQ1 application. In contrast, treatment of cells with 1 μmol/L daunorubicin caused a marked increase in γH2AX foci within 1 h (data not shown). In subsequent experiments, cells were treated with 0.25 μmol/L JQ1 for 24 h and immunolabeled for γH2AX and proliferating cell nuclear antigen (PCNA), a marker of S-phase cells. We found significant overlap between PCNA and immunolabeling with ∼90% of γH2AX-positive cells being double labeled (Fig. 2D).

We also carried out experiments on cells treated with 0.25 μmol/L JQ1 and 10 μmol/L ATM inhibitor KU60019 simultaneously for 24 h which had no effect on the induction of γH2AX. Likewise, cells simultaneously treated with JQ1 and caffeine (3 μmol/L) to inhibit the ATR-CHK1 pathway showed no reduction in the number of cells with γH2AX pan-nuclear labeling. Neither KU60019 nor caffeine alone stimulated the appearance of γH2AX. Blocking of DNA-PK with 1 μmol/L NU7026 did, however, prevent the nuclear accumulation of γH2AX in JQ1-treated cells.

### JQ1 induces apoptosis via a caspase 3/7 but not caspase 8-dependent mechanism

**Figure 3:**
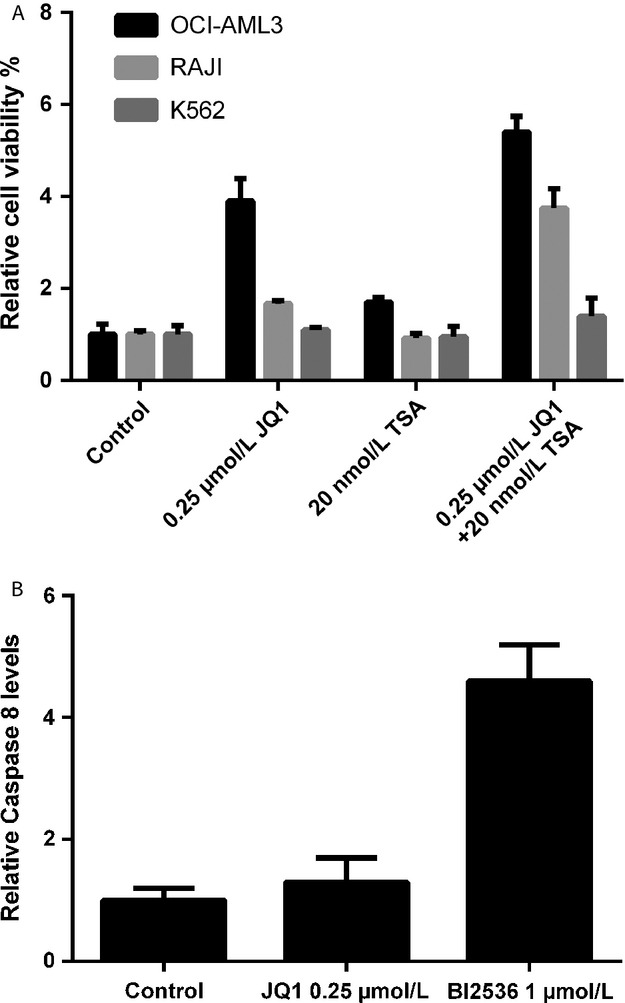
JQ1 activates caspase 3/7 but not caspase 8. (A) OCI-AML3, Raji, and K562 cells were plated at a density of 20,000 cells per well in a 96-well plate, treated with 0.25 μmol/L JQ1 for 24 h, then assayed for caspase3/7 activation in the presence or absence of 20 nmol/L TSA. (B) OCI-AML3 cells treated with 0.25 μmol/L JQ1 were assayed for caspase 8 activity (positive control experiment was performed using 1 μmol/L polo-like kinase inhibitor BI-2536).

Cells respond to DNA damage by activating signaling cascades that cause cell cycle arrest to allow repair or cause apoptosis to eliminate irreparably damaged cells. As caspases are essential in cells for apoptosis, we carried out experiments to determine if caspase activation occurs in OCI-AML3 cells treated with JQ1. Cells were treated with 0.25 μmol/L JQ1 for 24 h and the luminescent signal produced by caspase activation was measured using caspase 3/7 and caspase 8 Glo kits (Promega). Figure 3A shows that activation of caspase 3/7 occurs in JQ1-treated cultures by 24 h. However, activation was not seen at 2 or 4 h time points. Caspase 3/7 activation was also detected at 24 h in the Raji cell line but not in K562 cells that are poorly responsive to JQ1.

Caspase 8, a caspase activated by the extrinsic apoptotic pathway, was not activated by 24 h of JQ1 treatment but was activated by BI2536 a polo-like kinase inhibitor, as previously shown [13] (Fig. 3B).

### Synergistic studies involving JQ1 and other compounds

**Figure 4:**
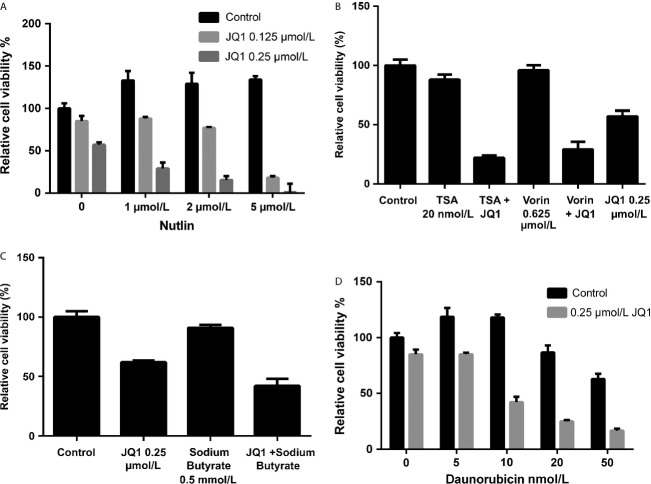
The effect of JQ1 on OCI-AML cells is enhanced by Nutlin-3, HDAC inhibitors, and daunorubicin. Cells were plated at a density of 20,000 cells per well in a 96-well plate and then treated with: (A) 0.25 μmol/L JQ1 plus 0–2 μmol/L Nutlin-3; (B) 0.25 μmol/L JQ1 plus either 20 nmol/L TSA or 0.625 μmol/L Vorinostat (vorin) for 72 h; (C) 0.25 μmol/L JQ1 plus 0.5 mmol/L sodium butyrate; (D) 0.25 μmol/L JQ1 plus 0–50 nmol/L daunorubicin. Cell viability was assayed using the WST-1 assay and results shown are from three separate experiments performed in triplicate ± SEM.

Caspase-dependent apoptosis is generally activated by p53-dependent signal transduction pathways. Therefore, we carried out experiments to test whether the p53 signal transduction pathway could be implicated in JQ1-induced apoptosis. We incubated cells with JQ1 together with Nutlin-3, an HDM2 inhibitor. Compounds were applied simultaneously to cells for 72 h. Nutlin-3 alone caused no decrease in cell viability (over range 1–5 μmol/L). However, in combination with 0.25 μmol/L JQ1, there was a marked decrease in cell viability that was significantly greater than for JQ1 alone (Fig. 4A). Pre-treatment of the cells with Nutlin-3 for 1 h before applying JQ1 produced the same outcome.

The WST-1 assay is not able to distinguish between effects on proliferation and cell death, so we hitherto employed annexin V/propidium iodide flow cytometry to directly measure apoptosis. Further experiments were carried out to determine the role of HDACs in the mechanism of action of JQ1 as HDAC inhibitors have been observed to activate p53 by acetylation [14]. We employed three HDAC inhibitors, namely TSA, sodium butyrate, and vorinostat, all of which exhibit activity against class I and II HDACs [14]. We found that all three HDAC inhibitors used at low concentrations potentiated the effect of JQ1 in reducing cell viability (Fig. 4B and C) and that for TSA this involved caspase 3/7-mediated apoptosis (Fig. 3A).

We carried out additional experiments with the anthracycline daunorubicin, a chemotherapy drug used in standard AML treatment regimes. Again we found synergy with this compound and JQ1 (Fig. 4D). Other compounds tested including the ATM inhibitor KU60019, cytosine arabinoside, polo-like kinase inhibitor BI2536, p38 inhibitor SB203580 and mTOR inhibitor RAD001, none of which showed evidence of synergy with JQ1 (data not shown).

### BRD4 interacts with p53 in OCI-AML3 cells

**Figure 5:**
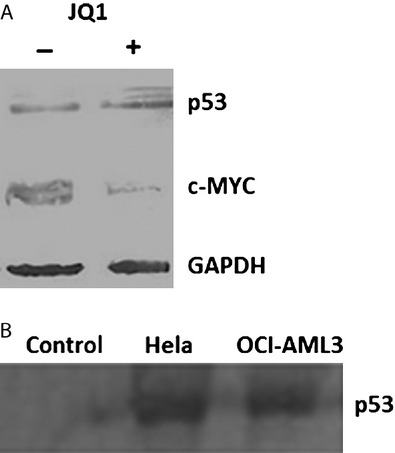
(A) Brd4 interacts with p53 in a JQ1-independent manner. Cells were treated with 0.5 μmol/L JQ1 for 24 h and then the extracted protein was subjected to gel electrophoresis and Western blotting for p53 and c-MYC (GAPDH was used as a loading control). (B) Co-immunoprecipitation studies were performed by immunoprecipitating the cell extracts for both Hela and OCI-AML3 cells with anti-BRD4 (or rabbit immunoglobulin as a negative control), and then Western blotting with anti-p53.

Our results indicate that the effects of JQ1 may be mediated at least partly by the p53 pathway. Therefore, we conducted experiments to investigate whether JQ1 had any effect on p53 protein expression. OCI-AML3 cells were incubated with 0.5 μmol/L JQ1 for 24 h and cell extracts were then subjected to Western blotting. Figure 5A shows that JQ1 had no effect on endogenous p53 levels. Blots were probed with anti-c-MYC as a positive control for JQ1 treatment. As expected, c-MYC levels were downregulated.

We hypothesized that BRD4 might interact with acetylated p53 via bromodomain binding. OCI-AML3 and Hela cells were exposed to daunorubicin and TSA in order to induce p53 expression and hyper-acetylation. Co-immunoprecipitation experiments were then performed on the cell extracts using anti-BRD4 antibodies and probing the Western blots with anti-p53. This revealed that BRD4 associates with endogenous acetylated p53 as hypothesized with no band seen in the rabbit immunoglobulin control lane (Fig. 5B). In order to determine if this association occurred through an acetyl-lysine-dependent mechanism, we repeated the co-immunoprecipitation experiment using protein extract from cells treated with 1 μmol/L JQ1 in addition to daunorubicin and TSA. We found that JQ1 exposure did not disrupt the association between BRD4 and p53 (data not shown). Similarly, JQ1 added to the extracted protein lysate at concentrations up to 1 mmol/L failed to disrupt the association between BRD4 and p53. On the basis of these observations, we conclude that BRD4 binds p53 in a bromodomain-independent manner that is not affected by JQ1. Rather, we propose that JQ1 prevents BRD4-mediated recruitment of p53 to chromatin thereby preventing normal activation of the DNA damage repair response and leading instead to cellular apoptosis.

## Discussion

Previous studies have shown that the BET bromodomain inhibitor JQ1 causes cell death of both leukemia and myeloma patient samples and of cell lines [1, 3, 4]. Here we present data relating to the action of JQ1 on the p53-wildtype OCI-AML3 leukemia cell line, which carries mutations of the DNMT3A and NPM1 genes that are known to occur frequently in AML. We show that the OCI-AML3 cell line is sensitive to submicromolar concentrations of JQ1. JQ1 induces a dose-dependent decrease in cell viability, caspase3/7-mediated apoptosis, and an increase in DNA damage. In addition, we show synergy between JQ1 and daunorubicin, HDAC inhibitors and the HDM2 inhibitor Nutlin-3 in causing apoptosis. JQ1-mediated apoptosis is stimulated by caspase 3/7 activation but not caspase 8 activation indicating that the intrinsic apoptotic pathway is involved.

JQ1 induces DNA double-stranded breaks (DSB) in OCI-AML3 cells as monitored by the appearance of 53BP1 foci, a gene that is critical for the control of DSB repair, and stimulates a massive upregulation in phosphorylated histone H2AX. Phosphorylation of H2AX at serine 139 (γH2AX) is an early sign of DNA damage induced by replication stalling. Upregulation takes at least 7 h and is maximal within 24 h. γH2AX is not detected as discrete foci but rather as uniform pan nuclear staining. This pattern of γH2AX distribution has been reported in previous studies [15, 16]. In both this study and that of de Feraudy et al. [15], a correlation between γH2AX and S-phase was observed.

The accumulation of pan nuclear γH2AX was not affected by ATM or ATR inhibitors but was reduced by DNA-PK inhibition. DNA-PK phosphorylates H2AX during apoptotic fragmentation in mammalian cells with a delayed time course relative to ionizing radiation [17]. Therefore, we conclude that in our cells γH2AX phosphorylation is induced by JQ1 in response to apoptotic fragmentation. DNA-PK signaling of DNA damage is probably mediated by p53 [18, 19].

Previous studies have demonstrated a clear role for c-MYC in the mechanism of action of JQ1. c-MYC levels are rapidly downregulated in many hematological cell lines [3, 4] confirmed in our Western blotting experiments on OCI-AML3 cells. Furthermore, CHIP experiments reveal that BRD4 binds to the c-MYC promoter, an action that is reversed by JQ1 [3, 4]. However, c-MYC downregulation alone is not sufficient to induce apoptosis as several cell lines (K562, Jurkat, and MDA MB-231) in which JQ1 induces very little cell death show significant downregulation of c-MYC transcripts [1, 20].

Our data suggest a role for p53 in the mechanism of action of BRD4. Numerous studies including ours have shown that BRD4 inhibition leads to cell cycle arrest, senescence, p21 upregulation and apoptosis, all processes mediated by p53 [21]. In addition, we show synergy between JQ1 and the HDM2 inhibitor Nutlin-3, HDAC inhibitors and the anthracycline daunorubicin. Nutlin-3 has been shown to rapidly activate p53 in cancer cells with wildtype p53 [22, 23] by inhibiting the interaction between HDM2 and p53, thus stabilizing p53. Likewise, we show that HDAC inhibitors, compounds that maintain p53 in an active acetylated form [24], synergize with JQ1. Acetylation of p53 is crucial for p53-mediated apoptosis [25] and also inhibits p53–HDM2 interactions [26, 27]. In addition, our results show that DNA-damaging agents such as daunorubicin, a chemotherapy agent commonly used in leukemia treatment, also synergize with JQ1 in OCI-AML3 cells. Daunorubicin causes p53 to bind to DNA [28] providing additional support for a role for p53 in JQ1-induced apoptosis.

Our Western blotting studies show that the effects of JQ1 are not mediated by a direct effect of BRD4 inhibition on p53 protein levels. However, our immunoprecipitation experiments show that p53 directly interacts with BRD4 in both the OCI-AML3 and Hela cell lines confirming the recently published observations of Wu et al. [29]. Further evidence for a role for p53 comes from gene expression analysis of acute lymphoblastic leukemia cells that revealed that genes involved in p53 stabilization are upregulated in response to JQ1 [30]. We show that the association of p53 with BRD4 is not inhibited by JQ1, suggesting it does not occur via a bromodomain-dependent mechanism. Indeed, it has very recently been reported that the interaction of BRD4 and p53 is modulated by two conserved regions, namely the phosphorylation-dependent interaction domain (PDID, that encompasses bromodomain 2) and the basic residue-enriched interaction domain (BID) [29]. Although the protein extracts used in our immunoprecipitation experiments were hyper-acetylated by prior exposure to TSA, we did not conclusively establish whether acetylation of p53 facilitates its association with BRD4. However, our observations clearly raise this possibility and are consistent with other studies that show interaction between purified BRD4 and acetylated p53 in vitro [29]. Other bromodomain-containing proteins have also been shown to interact with acetylated p53 including BRD7 [31] and p300/CBP [32, 33]. BRD4 itself has been shown to bind to acetylated NFκB [7]. As yet the effect of acetylation of p53 on BRD4 interactions and gene transcription has not been analyzed.

Interestingly, it has been shown that p53 can mediate transcriptional repression of c-MYC. p53 is required for c-MYC-dependent cell cycle arrest and differentiation but not apoptosis [34], a finding in concert with the actions of JQ1 where ectopic expression of c-MYC in hematological cell lines confers significant resistance to JQ1-induced cell cycle arrest and differentiation [1, 3] but cell death is not affected [1]. Importantly, JQ1 appears to be particularly potent against AML cells which usually lack p53 mutations occurring in less than 10% of patients [35] compared with around 50% for other human cancers [36].

In conclusion, this study suggests that inhibition of BRD4 by JQ1 induces cell cycle arrest and/or apoptosis of AML cells in a p53-mediated manner. We find that compounds known to induce p53 activity such as daunorubicin via DNA damage, Nutlin-3 via HDM2 inhibition, and TSA via HDAC inhibition, all result in significant synergy with JQ1 in OCI-AML3 cells, an AML cell line with intact functional p53. We show that p53 binds BRD4 but that this interaction is not disrupted by JQ1, implying that binding is not mediated by either of the two bromodomains. This is in agreement with the studies of Wu et al. [29]. We hypothesize that BRD4 is normally responsible for recruiting p53 to chromatin in response to DNA damage leading to cell cycle arrest and that this process is inhibited by JQ1 leading to cell death in a c-MYC-independent manner. In the case of AML, where p53 is commonly intact, these findings suggest that BET bromodomain inhibition could potentiate the activity of other chemotherapeutic agents that are known to act via p53 activation. Finally, we show that JQ1 is active against the leukemic cell line OCI-AML3, which contains the commonly recurring NPM1c mutation and the highly adverse DNMT3A R882C mutation, confirming its promise as a novel therapy in AML.
